# Seasonal and Environmental Influences on the Gut Microbiota of South China Tigers (*Panthera tigris amoyensis*)

**DOI:** 10.3390/ani15101471

**Published:** 2025-05-19

**Authors:** Li Zhou, Xiyao Xu, Zhirong Zhang, Xu Zhang, Kaixiong Lin, Hongxing Luo, Cheng Huang, Xipan Lin, Chunli Zhang, Yan Qing, Liwei Teng, Zhensheng Liu

**Affiliations:** 1 College of Wildlife and Protected Area, Northeast Forestry University, Harbin 150040, China; 2 Fujian Meihuashan Institute of South China Tiger Breeding, Longyan 364201, China; 3 Key Laboratory of Conservation Biology, National Forestry and Grassland Administration, Harbin 150040, China

**Keywords:** South China tiger, gut microbiota, fungi, captive conditions, seasonal variation

## Abstract

In ex situ conservation, gut bacteria and fungi play a crucial role in maintaining the intestinal microecological balance of the gut, and disruptions in this system may negatively impact host health. The South China tiger (*Panthera tigris amoyensis*) is a critically endangered tiger subspecies currently surviving under human-managed care and captive breeding programs, with only a small number of individuals remaining. Disruption in the gut microbiota, particularly the proliferation of pathogenic bacteria, can be fatal for cubs. This study analyzed the composition and seasonal changes of the gut bacterial and fungal communities of South China tigers in captive and semi-released environments during the winter of 2023 and the summer of 2024, using 16S rRNA and ITS high-throughput sequencing. The results showed that the dominant gut bacteria were Bacillota, Actinomycetota, Fusobacteriota, Pseudomonadota, and Bacteroidota. The abundance of Bacillota decreased in summer and was slightly lower in captive tigers than in semi-released tigers. Fungal communities were dominated by Ascomycota, with a negative correlation observed between Ascomycota and Basidiomycota. The abundance of Ascomycetes was lower in summer but significantly higher in semi-released tigers than in captive ones. Diversity analysis revealed no significant effects of the season or the environment on bacterial α-diversity, and fungal α-diversity was also not significantly affected by seasonal variation. However, microbial diversity increased under semi-free-range conditions, with fungal richness significantly higher than in captive environments (*p* < 0.01). Principal coordinate analysis (PCoA) based on Bray–Curtis distances revealed significant differences in the community structures of both bacterial (Adonis, R^2^ = 0.2364, *p* = 0.001) and fungal (Adonis, R^2^ = 0.1542, *p* = 0.001) communities across different seasons and environments. These findings provide valuable insights into the health management strategies and ecological adaptation of South China tigers.

## Simple Summary

The South China tiger (*Panthera tigris amoyensis*), a critically endangered subspecies, currently depends entirely on human-provided feeding for survival. The gut microbiota plays a crucial role in digestion, immune function, and overall health. However, its response to varying captive environments and seasonal changes remains unclear. In this study, we analyzed the gut bacterial and fungal communities of South China tigers in fully captive and semi-free-ranging conditions across different seasons. The dominant bacterial phyla identified were Bacillota, Actinomycetota, Fusobacteriota, Pseudomonadota, and Bacteroidota, while the fungal communities were primarily composed of Ascomycota and Basidiomycota. Our results indicated that bacterial diversity remained relatively stable, whereas fungal communities were more affected by the environment. Semi-free-ranging tigers exhibited a more balanced gut microbiota, suggesting potential health benefits. These findings provide insights into optimizing feeding and management strategies to improve the health and conservation prospects of this endangered species.

## 1. Introduction

As a flagship species for global biodiversity conservation, the South China tiger (*Panthera tigris amoyensis*) holds profound ecological and cultural importance [1,2]. Ecologically, apex predators like tigers regulate prey populations and maintain the structural integrity of their ecosystems, preventing overgrazing and promoting biodiversity [3]. Historically, the South China tiger ranged throughout Central and Southern China, playing a vital role in forest ecosystem dynamics [4]. Unfortunately, it is the only tiger subspecies that now survives exclusively in captivity due to human-induced pressures such as habitat fragmentation and historical overhunting [5,6]. In contrast to other subspecies that still exist in the wild, the South China tiger faces unique conservation challenges, particularly in terms of genetic bottlenecks and behavioral adaptation for potential reintroduction [4,7]. Reintroduction programs serve as a crucial strategy for restoring wild populations, and the gut microbiome is viewed as a key monitoring indicator for the success of captive reintroductions [8,9]. However, the success of these programs depends on a comprehensive understanding of the factors that influence tiger health and adaptation. Although previous studies have mainly focused on genetics [6,10,11,12], reproduction [13], and disease management [14,15,16], there remains a critical gap in knowledge regarding the role of gut microbiota, particularly under different environmental and seasonal conditions, and how they shape physiological resilience and survival potential.

Studies on South China tigers have demonstrated that the composition and structure of their gut microbiota are influenced by age, developmental stage, and health status [17,18,19]. For example, captive South China tiger cubs exhibit marked shifts in gut bacterial composition, characterized by an increase in Bacillota and a decrease in *Thermodesulfobacteria*, before reaching a stable state in adulthood [18,20]. South China tigers suffering from diarrhea display significantly reduced intestinal bacterial abundance compared to healthy individuals [17]. However, these findings were derived under uniform, controlled captive conditions and thus fail to account for environmental variability. Importantly, no studies have yet investigated how different captivity conditions, such as enclosed cages versus semi-free-ranging environments, or seasonal variations influence the gut microbial communities of South China tigers. Environmental and seasonal variations are well known to affect the gut microbial composition of mammals. For example, wild Siberian tigers (*Panthera tigris altaica*) exhibit distinct bacterial diversity compared to their captive counterparts [21]. Sumatran tigers (*Panthera tigris sumatrae*) maintain relatively stable microbial communities across seasons, but individual responses to environmental changes can vary [22]. These differences underscore the sensitivity of the gut microbiome to external factors. However, the underlying mechanisms driving such shifts in endangered felids remain poorly understood.

The gut microbiome, comprising bacteria, fungi, archaea, and viruses, plays a critical role in host metabolism, immune function, and stress regulation [22,23]. Although most research has traditionally focused on bacterial communities, increasing evidence over recent years highlights the fungal microbiota as an equally important component, particularly in immune regulation and disease pathogenesis [24,25]. In the gut, fungi closely interact with bacteria and the host’s mucosal immune system, contributing significantly to the maintenance of intestinal homeostasis. They occupy similar ecological niches on the intestinal mucosal surface, and dysbiosis in fungal communities can lead to alterations in bacterial composition [26]. In tigers, host genetics and diet significantly shape fungal beta diversity, while age has minimal effects [27]. Disruptions in fungal homeostasis are associated with gastrointestinal disorders in mammals, emphasizing their potential relevance to the health of captive animals [25]. Additionally, certain fungi, such as *Candida albicans*, *Aspergillus*, and *Meyerozyma*, have been identified as potential pathogens in metabolic diseases due to their capacity to activate the immune system and produce harmful metabolites. Conversely, genera like *Saccharomyces*, *Kluyveromyces*, *Aureobasidium*, and *Wallemia* may exert beneficial effects on metabolic health [28]. However, environmental factors driving fungal dynamics, such as enclosure microclimate and seasonal dietary changes, remain unexplored in endangered cats. This gap hinders the comprehensive evaluation of microbial-mediated adaptation and poses a major challenge to improving captive management and pre-reintroduction regulation strategies.

This study aims to address two primary questions: (i) How do captive and semi-free-ranging environments influence gut bacterial and fungal communities in South China tigers during a single season? (ii) Do seasonal transitions from winter to summer induce measurable microbial restructuring under consistent rearing conditions? By integrating bacterial and fungal analyses, we seek to establish baseline data on environment–microbiome interactions, elucidate seasonal plasticity, and identify biomarkers associated with adaptive capacity. Our findings offer direct guidance for captive breeding centers to design habitat simulation strategies that restore the natural behavioral abilities of the South China tiger and enhance its wild instincts, which is a critical step in reversing the extinction trajectory of this iconic subspecies.

## 2. Materials and Methods

### 2.1. Sample Collection

At the Fujian Meihuashan Institute of South China Tiger Breeding in China, two primary housing environments were utilized for tiger management. One was an indoor captive setting, where individuals were kept on cement or wooden floors. The other was an outdoor semi-free-ranging enclosure, characterized by abundant natural vegetation, including evergreen broad-leaved shrubs, grasses, and bamboo, as well as access to water sources and natural or artificial caves. Tigers in both environments received a standardized diet comprising beef, chicken, and farm-raised wild boar, with feeding quantities maintained consistently across groups. However, the semi-free-ranging environment provided additional access to natural vegetation, and individuals were occasionally observed foraging for herbaceous plants. This dietary variation induced by the environment may indirectly influence gut microbiota composition. All sampled individuals in the captive group were housed individually. In the semi-free-ranging group, some individuals were occasionally co-housed. To ensure accurate individual sampling, co-housed tigers were identified by their stripe patterns and separated 1 day before fecal collection.

To ensure the freshness of fecal samples, the following criteria were applied: a smooth and moist surface, no foreign materials, and no signs of dehydration or decomposition. For captive individuals, feces excreted overnight or in the early morning were collected before enclosure cleaning. Samples that appeared dry or were suspected to have been excreted the previous day were excluded. For semi-free-ranging tigers, target individuals were lured into an isolated enclosure the day before sampling. On the collection day, the tiger was guided into a holding area, allowing researchers to enter the vacated enclosure to locate and collect fresh feces. In some cases, individuals were temporarily transferred into indoor housing; once defecation was observed, the tiger was returned to the semi-free-ranging environment, and researchers subsequently entered to retrieve the fresh sample.

Fresh fecal samples were collected from five captive and six semi-free-ranging adult South China tigers in December 2023 (winter) and again from the same individuals in July 2024 (summer). Each individual was sampled three times per season at 1-week intervals, resulting in 15 samples from the winter captive group (C-WT), 18 from the winter semi-free-ranging group (SF-WT), 15 from the summer captive group (C-SM), and 18 from the summer semi-free-ranging group (SF-SM). During sample collection, researchers wore sterile gloves and transferred the central portion of each fecal sample into a 100 mL sterile centrifuge tube, recording the individual identity and time of collection. All samples were immediately stored at −80 °C and transported to the laboratory on sufficient dry ice after the completion of seasonal sampling for subsequent analysis.

### 2.2. DNA Extraction and Sequencing

Genomic DNA was extracted from all fecal samples using the FastPure Stool DNA Isolation Kit (Magnetic bead) (MJYH, Shanghai, China), and DNA quality was assessed via 1% agarose gel electrophoresis. For bacterial community analysis, the V3-V4 region of the 16S rRNA gene was amplified using primers 338F (5′-ACTCCTACGGAGGCAGCAG-3′) and 806R (5′-GGACTACHVGGGTWTCTAAT-3′) [29]. For fungal community analysis, the ITS1 region of the fungal ITS rRNA gene was amplified using primers ITS1F (5′-CTTGGTCATTTAGAGGAAGTAA-3′) and ITS2 (5′-GCTGCGTTCTTCATCGATGC-3′) [18]. The PCR amplification reaction system included 10 μL 2× Pro Taq premix, 0.8 μL forward primer (5 μM), 0.8 μL reverse primer (5 μM), and 10 ng/μL template DNA, with the final volume adjusted to 20 μL. PCR reaction conditions were as follows: initial denaturation at 95 °C for 3 min; 29 (for 16S) or 35 (for ITS) cycles of denaturation at 95 °C for 30 s, annealing at 53 (for 16S) or 55 °C (for ITS) for 30 s, and extension at 72 °C for 30 s. A final extension was performed at 72 °C for 10 min, and samples were held at 10 °C until further processing (PCR instrument: ABI GeneAmp^®^ 9700, Applied Biosystems, Foster City, CA, USA). Following verification, gel recovery, and purification, qualified samples were subjected to high-throughput sequencing on the Illumina MiSeq PE300 platform (Shanghai Meiji Biomedical Technology Co., Ltd., Shanghai, China).

### 2.3. Data Processing

Pre-processing, quality control, and sequence filtering of raw sequencing data were conducted using the Majorbio Cloud platform (https://cloud.majorbio.com; accessed on 5 March 2025). Operational taxonomic units (OTUs) were clustered at a sequence similarity threshold of 97%, with chimeric sequences removed during the clustering process. Taxonomic annotation was performed using the Bayesian RDP classifier algorithm (version 2.11) [30]. OTUs for 16S rRNA sequences were assigned based on the SILVA database (version 138), while OTUs for ITS1 sequences were classified using the UNITE database (version 7.2).

For bacterial analysis, a total of 3,555,042 high-quality sequences were obtained, comprising 1,446,938,802 bases with an average sequence length of 407 bp. A total of 1565 OTUs were identified and classified into 25 phyla, 53 classes, 133 orders, 252 families, and 522 genera. The rarefaction curves (Figure S1) plateaued, indicating that sequencing depth was sufficient and no additional OTUs would likely be discovered. Similarly, the species accumulation curves (Figure S2) reached saturation, confirming adequate sample size and sequencing depth.

For fungal analysis, a total of 4,738,842 optimized sequences were obtained, comprising 1,081,777,949 bases with an average sequence length of 228 bp. A total of 3976 OTUs were identified, classified into 15 phyla, 63 classes, 166 orders, 421 families, and 915 genera. While most rarefaction curves (Figure S3) reached plateaus, the curves for group C did not fully flatten, suggesting the possible presence of rare, undetected OTUs despite generally adequate sequencing depth. The species accumulation curves (Figure S4) also plateaued, confirming that the sequencing depth and sample size were appropriate.

### 2.4. Statistical Analyses

All data analyses were conducted using R software (version 4.4.3) in conjunction with the Majorbio Cloud platform. The 10 most abundant bacterial and fungal phyla and the 20 most abundant bacterial and fungal genera were analyzed at their respective taxonomic levels. The R packages “phyloseq” and “vegan” were employed to calculate α-diversity indices at the OTU level, including Chao, ACE, Shannon, and Simpson indices (http://www.mothur.org/wiki/Calculators; accessed on 5 March 2025) [31]. Group-level differences in α-diversity were assessed by first applying the Shapiro–Wilk test to evaluate the normality of each index. For indices that conformed to a normal distribution, one-way analysis of variance (ANOVA) was performed, followed by Tukey–Kramer post hoc tests for pairwise comparisons (Table S1). For the non-normally distributed Chao, ACE, and Simpson indices, the Kruskal–Wallis rank-sum test was used, followed by Dunn’s post-hoc test for pairwise comparisons. Principal Coordinates Analysis (PCoA) based on Bray–Curtis dissimilarity was conducted to evaluate β-diversity at the OTU level. Differences in microbial community composition among groups were assessed using permutational multivariate analysis of variance (PERMANOVA) via the adonis function in the vegan package, with *p*-values adjusted for multiple comparisons. All β-diversity analyses and visualizations were conducted using the phyloseq and vegan packages. To identify taxa significantly contributing to community differences, linear discriminant analysis effect size (LEfSe) analysis was performed using the Majorbio Cloud platform. The analysis was conducted across taxonomic levels from phylum to genus, with a logarithmic LDA score threshold of >4 [32]. Functional predictions for bacterial and fungal communities were conducted using PICRUSt2 and FUNGuild, respectively, also on the Majorbio Cloud platform [33,34].

## 3. Results

### 3.1. Bacterial and Fungal Communities in the Gut of South China Tigers

**Figure 1:**
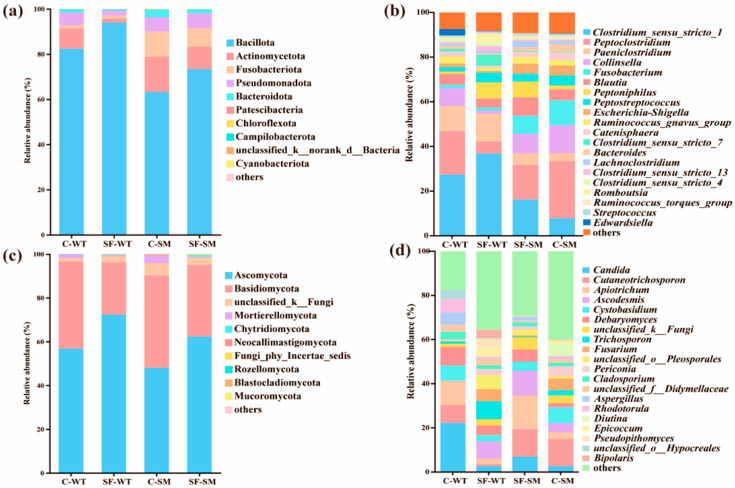
Relative abundance of gut microbiota in South China tigers at the phylum and genus levels. (**a**) Composition of bacterial communities at the phylum level. (**b**) Composition of bacterial communities at the genus level. (**c**) Composition of fungal communities at the phylum level. (**d**) Composition of fungal communities at the genus level. Different colors represent different microbial taxa.

Bacillota, Actinomycetota, Fusobacteriota, Pseudomonadota, and Bacteroidota collectively accounted for over 99% of the total bacterial abundance across all groups, representing the dominant bacterial phyla in South China tigers (Figure 1a, Table S2). The five most abundant bacterial genera were *Clostridium_sensu_stricto_1*, *Peptoclostridium*, *Paeniclostridium*, *Collinsella*, and *Fusobacterium* (Figure 1b, Table S3). The dominant fungal phyla were Ascomycota and Basidiomycota, with their combined abundance exceeding 90% in all groups (Figure 1c, Table S4). At the genus level, the most abundant fungal genera were *Candida*, *Cutaneotrichosporon*, *Apiotrichum*, *Ascodesmis*, *Cystobasidium*, and *Debaryomyces*. Except for *Cystobasidium*, notable variations were observed among the groups. The abundance of *Candida* and *Apiotrichum* was higher in the C groups than in the SF groups, with minimal differences between the SF-WT and SF-SM groups. Within the C groups, *Candida* abundance was higher in the C-WT group than in the C-SM group, whereas *Apiotrichum* showed the opposite trend (Figure 1d, Table S5).

### 3.2. Effects of Environment and Seasonal Changes on Bacterial and Fungal Diversity

**Figure 2:**
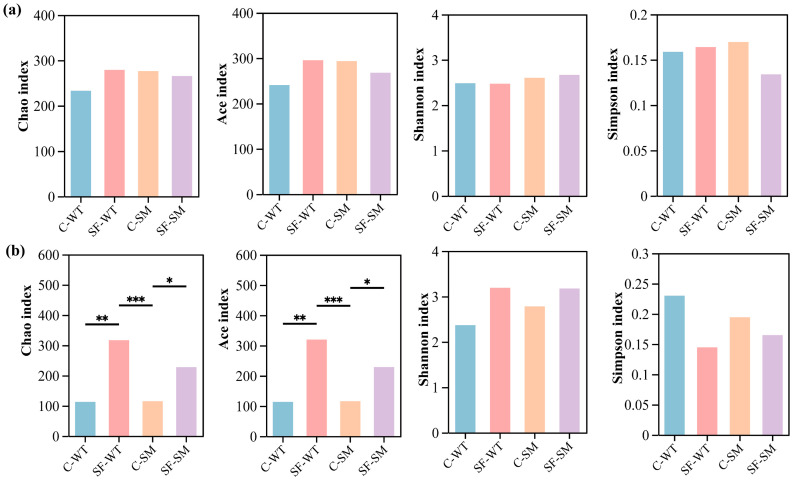
Alpha diversity of intestinal microbiota in South China tigers. (**a**) Bacterial alpha diversity indices. (**b**) Fungal alpha diversity indices; diversity was assessed using Chao, ACE, Shannon, and Simpson indices. Asterisks indicate levels of statistical significance: *p* < 0.05 (*), *p* < 0.01 (**), and *p* < 0.001 (***).

**Figure 3:**
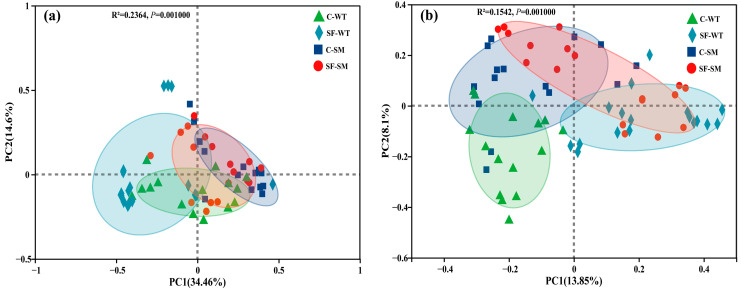
Principal coordinate analysis (PCoA) of bacterial and fungal gut microbiota in South China tigers based on Bray–Curtis distances. (**a**) PCoA plot of bacterial communities; (**b**) PCoA plot of fungal communities. Each point represents the gut microbiota of an individual fecal sample. Different colors and shapes indicate different sample groups. Shorter distances between points indicate greater similarity in microbial community composition.

There were no significant differences in the bacterial α-diversity indices among the groups (*p* > 0.05), suggesting that species richness and overall diversity were generally comparable across groups (Figure 2a). In contrast, fungal communities showed significant variation in richness across housing environments. Both the Chao and ACE indices were significantly higher in the SF group than in the C group during both winter and summer (*p* < 0.01) (Figure 2b), indicating greater fungal richness under semi-free-ranging conditions. Although no significant differences were observed in evenness, the Shannon index was higher and the Simpson index was lower in the SF group compared to the C group, both indicating increased fungal diversity under semi-free-ranging conditions (Figure 2b). PCoA based on Bray–Curtis distances at the OTU level shows that PC1 and PC2 explained 34.46% and 14.6% (Figure 3a) of the variance in the bacterial community, respectively, while in the fungi, 13.85% and 8.1% (Figure 3b) of the variance were explained. The ordinations revealed significant differences in microbial community structure between different seasons and environments, in both bacterial (Adonis, R^2^ = 0.2364, *p* = 0.001) (Figure 3a, Table S6) and fungal communities (Adonis, R^2^ = 0.1542, *p* = 0.001) (Figure 3b, Table S7).

### 3.3. Effects of Environment and Seasonal Changes on the Dominant Gut Microbiota of South China Tigers

**Figure 4:**
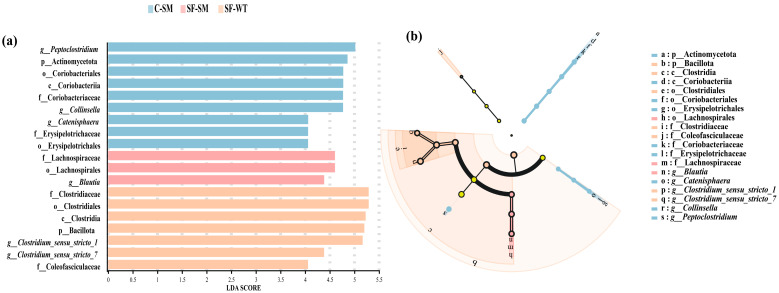
Differential analysis of intestinal bacterial communities in South China tigers based on (linear discriminant analysis effect size) LEfSe. (**a**) Bar chart of log-transformed LDA scores, showing bacterial taxa with significant differences among groups. (**b**) Cladogram illustrating the phylogenetic relationships of differentially abundant bacterial taxa. Different colors indicate distinct groups, but light yellow nodes represent microbial taxa that show no significant differences among different groups or have no significant impact on intergroup variation. Each circle represents a taxonomic level from phylum to genus (OTU), with circle size corresponding to relative abundance.

LEfSe analysis revealed significant differences in bacterial taxa across two phyla, two classes, four orders, five families, and six genera. No significantly enriched taxa were observed in the C-WT group. In the SF-WT group, seven taxa showed significant enrichment, notably Clostridiaceae, Clostridiales, Clostridia, Bacillota, and *Clostridium_sensu_stricto_1*. In the C-SM group, nine taxa, including *Peptoclostridium*, Actinomycetota, and Coriobacteriaceae, were significantly enriched. In the SF-SM group, three taxa, such as Lachnospiraceae, Lachnospirales, and *Blautia*, were prominent (Figure 4a). Apart from the taxa mentioned above, no additional differentially enriched taxa were detected in the cladogram analysis (Figure 4b).

**Figure 5:**
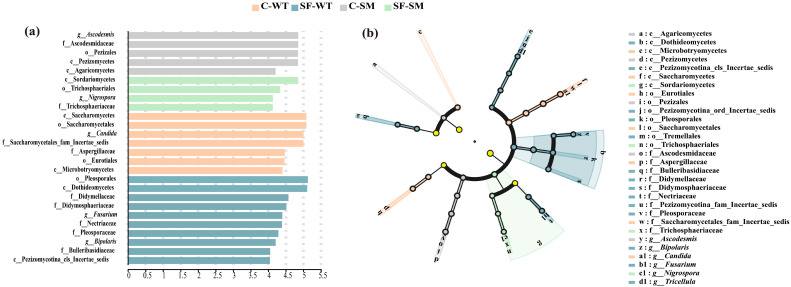
Differential analysis of intestinal fungal communities in South China tigers based on LEfSe. (**a**) Bar chart of log-transformed LDA scores, showing fungal taxa with significant differences among groups. (**b**) Cladogram illustrating the phylogenetic relationships of differentially abundant fungal taxa. Different colors indicate distinct groups, but light yellow nodes represent microbial taxa that show no significant differences among different groups or have no significant impact on intergroup variation. Each circle represents a taxonomic level from phylum to genus (OTU), with circle size corresponding to relative abundance.

For the fungal community, LEfSe analysis identified significant taxonomic differences among groups (Figure 5a), including seven classes, five orders, nine families, and five genera. In the C-WT group, Saccharomycetes, Saccharomycetales, *Candida*, and Saccharomycetales_fam_Incertae_sedis were the most distinct taxa. The SF-WT group displayed the greatest diversity of differential taxa, with Pleosporales and Dothideomycetes being the most prominent. In the C-SM group, *Ascodesmis*, Ascodesmidaceae, Pezizales, and Pezizomycetes were the most enriched. The SF-SM group had the fewest differential taxa, with Sordariomycetes and Trichosphaeriales being the most notable. The cladogram (Figure 5b) further highlighted significant enrichment of Pezizomycotina_ord_Incertae_sedis, Tremellales, Pezizomycotina_fam_Incertae_sedis, and *Tricellula* in the SF-WT group.

### 3.4. Prediction of Bacterial and Fungal Functions in the Gut of South China Tigers

**Figure 6:**
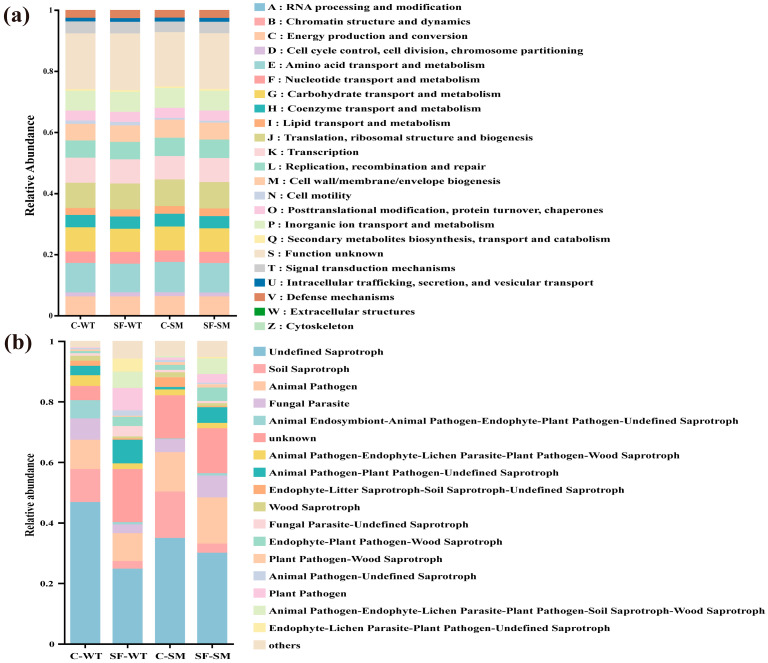
Predicted functional profiles of the gut microbiota in South China tigers. Predicted functional profiles of the gut microbiota in South China tigers. (**a**) Bacterial functions predicted using (Clusters of Orthologous Groups) COG classification. (**b**) Fungal ecological functions inferred through FUNGuild.

Based on Clusters of Orthologous Groups (COG) classification, the functional prediction of gut bacterial communities revealed a broadly similar composition across all groups. The dominant functions were related to amino acid and carbohydrate metabolism, transcription, translation, and energy production, underscoring an active microbial role in nutrient processing and host interactions. Additionally, functions associated with replication and repair, cell wall biogenesis, and signal transduction were present, reflecting microbial adaptability and structural maintenance. The overall similarity in functional profiles suggests that, despite potential taxonomic differences, the core metabolic functions of the gut microbiota remain relatively stable across environments and seasons (Figure 6a).

For the fungal community, the predicted functional groups were predominantly saprotrophic and pathotrophic. Among them, the most abundant categories included undefined saprotrophs, soil saprotrophs, animal pathogens, fungal parasites, and a mixed group consisting of animal endosymbionts, endophytes, plant pathogens, and undefined saprotrophs. Notably, undefined and soil saprotrophs were more abundant in the C groups, while animal pathogens were slightly more prevalent in the SM groups compared to the WT groups (Figure 6b).

## 4. Discussion

### 4.1. Dynamics and Diversity of Intestinal Bacterial Communities in South China Tigers

As a critically endangered species, understanding the composition and dynamic abundance of intestinal microbiota in South China tigers offers crucial insights for disease prevention, diagnosis, treatment, and health management in captive populations. Due to the relatively low abundance of gut fungi, most previous studies on animal gut microbiota have focused primarily on bacterial communities [35], with limited attention provided to fungal components. However, intestinal fungi play a significant role in shaping gut function and influence the physiological status of various extraintestinal organs [36]. For example, a study of Bengal tigers demonstrated that abnormal proliferation of specific intestinal fungal hyphae could trigger abdominal sepsis, leading to clinical symptoms such as vomiting and diarrhea. This study employed 16S rRNA and ITS high-throughput sequencing technologies to investigate how different environmental conditions and seasonal variations on the composition and abundance of bacterial and fungal communities in the gut microbiota of South China tigers.

The dominant bacterial phyla identified in the intestines of South China tigers—Bacillota, Actinomycetota, Fusobacteriota, Pseudomonadota, and Bacteroidota—were consistent with those reported in previous studies on Bengal tigers, Siberian tigers [27], and Malayan tigers (*Panthera tigris jacksoni*) [37], suggesting that these phyla may play essential roles in maintaining intestinal homeostasis and metabolic functions across tiger subspecies. In contrast, distinct dominant bacterial phyla were observed in African lions (*Panthera leo*) [38], North China leopards (*Panthera pardus japonensis*) [39], and snow leopards (*Panthera uncia*) [40], likely due to interspecific differences in diet, physiology, and habitat. At the genus level, the dominant bacterial genera in South China tigers were *Clostridium_sensu_stricto_1*, *Peptoclostridium*, *Collinsella*, and *Fusobacterium*. A study conducted on three tiger subspecies at Guangzhou Zoo [27] found that the dominant genera in South China tigers, Siberian tigers, and Bengal tigers reported partial overlap with our findings. For example, the dominant genera in Siberian tigers included *Fusobacterium* and *Collinsella*, and similarly, the dominant genera in Malayan tigers [37] also included *Collinsella*, *Clostridium_sensu_stricto_1*, and *Fusobacterium*. These results support the conclusion proposed by Jiang et al. that the gut microbiota of tigers has not significantly changed across different subspecies [27]. The observed differences in dominant genera across studies may be attributed to geographic variation and differences in feeding strategies.

Although the environmental conditions and seasonal variations influence bacterial abundance, they do not alter the dominant phyla. Bacillota, a dominant phylum in the intestines of most terrestrial mammals [41], exhibited higher relative abundance in semi-free-ranging groups (SF) compared to captive groups (C), and in winter (WT) compared to summer (SM) groups. The highest abundance was observed in the SF-WT group, suggesting that both environmental exposure and seasonal shifts contribute to its enrichment. Studies on Siberian tigers have reported higher levels of Bacillota in wild individuals than in those in captivity, likely due to differences in diet and environmental pressures [21]. Bacillota plays a key role in fiber digestion within the host gut [42]. The consistent increase in Bacillota under naturalistic conditions observed in both South China and Siberian tigers indicates a shared ecological response. The semi-free-ranging environment, which more closely mimics natural conditions, allows South China tigers to ingest plant materials that are typically unavailable in captive settings, potentially explaining the elevated abundance of Bacillota. A similar seasonal trend has been reported in African lions, with higher Bacillota abundance during winter than summer [38], which is consistent with our observations in South China tigers. Bacillota is closely associated with energy metabolism and the production of short-chain fatty acids (SCFAs) [43]. Its increased abundance may reflect elevated metabolic demands in semi-free-ranging tigers, particularly for thermoregulation during colder winter periods [44].

Although bacterial community diversity (alpha diversity) remained relatively stable across all groups, significant differences in bacterial community structures were observed, underscoring the influence of environmental conditions and seasonal variations on microbial composition. Similar patterns have been reported in Siberian tigers [37] and Malayan tigers [21], where wild and captive individuals exhibited distinct microbial profiles. Environmental enrichment has been shown to promote beneficial gut bacteria and improve microbial balance. In this study, potentially beneficial genera such as *Lachnoclostridium* (with anti-inflammatory properties) and *Romboutsia* (a known probiotic) [45] were enriched in semi-free-ranging tigers, although at low abundance levels, suggesting that enhanced enrichment strategies may yield microbiological health benefits. Future research should explore how microbiota changes affect health and develop strategies to optimize gut microbial composition in South China tigers.

### 4.2. Intestinal Fungal Community Structure and Its Changes in South China Tigers

Although fungi represent a minor component of the intestinal microbiota, they play essential roles in maintaining gut homeostasis and modulating immune responses [26]. Research investigating the influence of environmental and seasonal factors on the gut mycobiome remains limited. In the present study, the dominant fungal phyla in the intestines of South China tigers were Ascomycota and Basidiomycota, consistent with other felids [27,46]. A negative correlation between these two phyla was observed, and the Basidiomycota/Ascomycota (B/A) ratio has been linked to inflammatory bowel disease (IBD) [47,48,49]. Higher B/A ratios indicate a greater abundance of pro-inflammatory fungi and may reflect increased IBD risk. In this study, Ascomycota abundance was higher in semi-free-range (SF) groups, while Basidiomycota was more abundant in captive (C) groups, indicating environmental influences. Captive enclosures are typically subject to daily cleaning and more frequent disinfection compared to semi-free-ranging environments. Moreover, in the semi-free-ranging setting, tigers were observed consuming herbaceous plants. These environmental and behavioral differences may act as key drivers shaping the gut fungal communities of South China tigers. Elevated B/A ratios in summer were particularly notable in the captive groups, potentially indicating increased IBD susceptibility during this season. The dominant fungal genera included *Candida*, *Cutaneotrichosporon*, *Apiotrichum*, *Ascodesmis*, *Cystobasidium*, and *Debaryomyces*. *Candida*, a common fungal pathogen, was more abundant in captive tigers and showed significant seasonal fluctuations, suggesting that the environmental factors play a major role in their prevalence, with the semi-free-ranging environment offering greater microbial stability.

Fungal diversity was higher in the SF groups during winter, while diversity remained relatively stable throughout the year in the C groups. Environmental factors had a more pronounced impact on fungal community structure than seasonal changes. These findings underscore that environmental and seasonal factors shape the diversity and composition of fungal communities in ways distinct from bacterial communities. This study provides new insights into how ecological context and temporal changes affect the intestinal fungal communities of South China tigers.

In future conservation and management strategies for captive populations, greater emphasis should be placed on the long-term effects of the housing environment on the intestinal microbiota. For example, optimizing the captive environment and enhancing dietary diversity may help sustain the richness and stability of both bacterial and fungal communities. Additionally, long-term microbial monitoring during feeding should be implemented, with feeding strategies adjusted in response to microbial shifts. Such approaches could enable more targeted health interventions and effective disease prevention. These efforts are essential for improving the health of captive South China tigers, enhancing their natural behaviors and wild instincts, and supporting the long-term conservation of the species. Nevertheless, future research should expand the sample size, incorporate more naturalistic environmental conditions, and integrate genomic approaches to better elucidate the functional roles of gut microbes. This will provide a scientific basis for disease prevention, precision health management, and dietary optimization in South China tigers.

## 5. Conclusions

This study examined the composition and temporal dynamics of intestinal bacterial and fungal communities in South China tigers under varying environmental and seasonal conditions. The findings revealed that, although the dominant bacterial phyla remained consistent across conditions, certain potentially beneficial bacterial taxa exhibited higher abundance in semi-free-ranging environments, potentially enhancing the tigers’ adaptability to natural habitats. Notably, bacterial and fungal communities displayed differential responses to environmental and seasonal factors. Fungal richness was significantly greater in semi-free-ranging settings compared to captive ones, whereas bacterial alpha diversity remained relatively stable. Both environmental conditions and seasonal variation significantly influenced the overall structure of the gut microbial communities.

Overall, these findings provide new insights into the health management and physiological adaptation of the South China tiger, highlighting the role of the gut microbiome in responding to environmental and seasonal changes. This study contributes to a deeper understanding of microbiome-mediated adaptation, crucial for optimizing captive management and restoring natural adaptability. Future research should further explore the functional significance of these microbial variations and their potential impacts on tiger health and adaptation.

## Data Availability

The datasets presented in this article are not readily available because the data are part of an ongoing study. Requests to access the datasets should be directed to corresponding author.

## Supplementary Materials

The following supporting information can be downloaded at: https://www.mdpi.com/article/10.3390/ani15101471/s1, Figure S1. The bacterial rarefaction curves. Figure S2. Bacterial rank abundance curves. Figure S3. The Fungal rarefaction curves. Figure S4. Fungal rank abundance curves. Table S1. Table X Shapiro–Wilk Test for normality of α-diversity indices (bacteria and fungi). Table S2. Top 10 enterobacterial phyla in terms of abundance in different combinations of seasons and environments. Table S3. Top 20 Enterobacteriaceae genera in abundance in different seasonal and environmental combinations. Table S4. Top 10 intestinal fungal phyla in terms of abundance in different combinations of seasons and environments. Table S5. Top 20 genera of enteric fungi in terms of abundance in different seasons and environmental combinations. Table S6. Intergroup comparison results for bacteria based on Adonis (with FDR correction). Table S7. Intergroup comparison results for fungi based on Adonis (with FDR correction).
