# Supplementary material for: Seasonal and Environmental Influences on the Gut Microbiota of South China Tigers (Panthera tigris amoyensis)
Source: Animals (Basel). 2025 May 19;15(10):1471. doi: 10.3390/ani15101471 (PMC12108431; doi:10.3390/ani15101471)
Supplement: Supplementary file 1 [file animals-15-01471-s001.zip › animals-3591978-supplementary.pdf]

Seasonal and Environmental Influences on the Gut Microbiota of South China Tigers (*Panthera tigris amoyensis*)

Li Zhou [1], Xiyao Xu [1], Zhirong Zhang [1], Xu Zhang [1], Kaixiong Lin [2], Hongxing Luo [2], Cheng Huang [2], Xipan Lin [2], Chunli Zhang [1], Yan Qing [1], Liwei Teng [1,3,*] and Zhensheng Liu [1,3,*]

1 College of Wildlife and Protected Area, Northeast Forestry University, Harbin 150040, China

2 Fujian Meihuashan Institute of South China Tiger Breeding, Longyan 364201, China

3 Key Laboratory of Conservation Biology, National Forestry and Grassland Administration, Harbin 150040, China

* Correspondence: tenglw1975@163.com (L.T.); zhenshengliu@163.com (Z.L.)

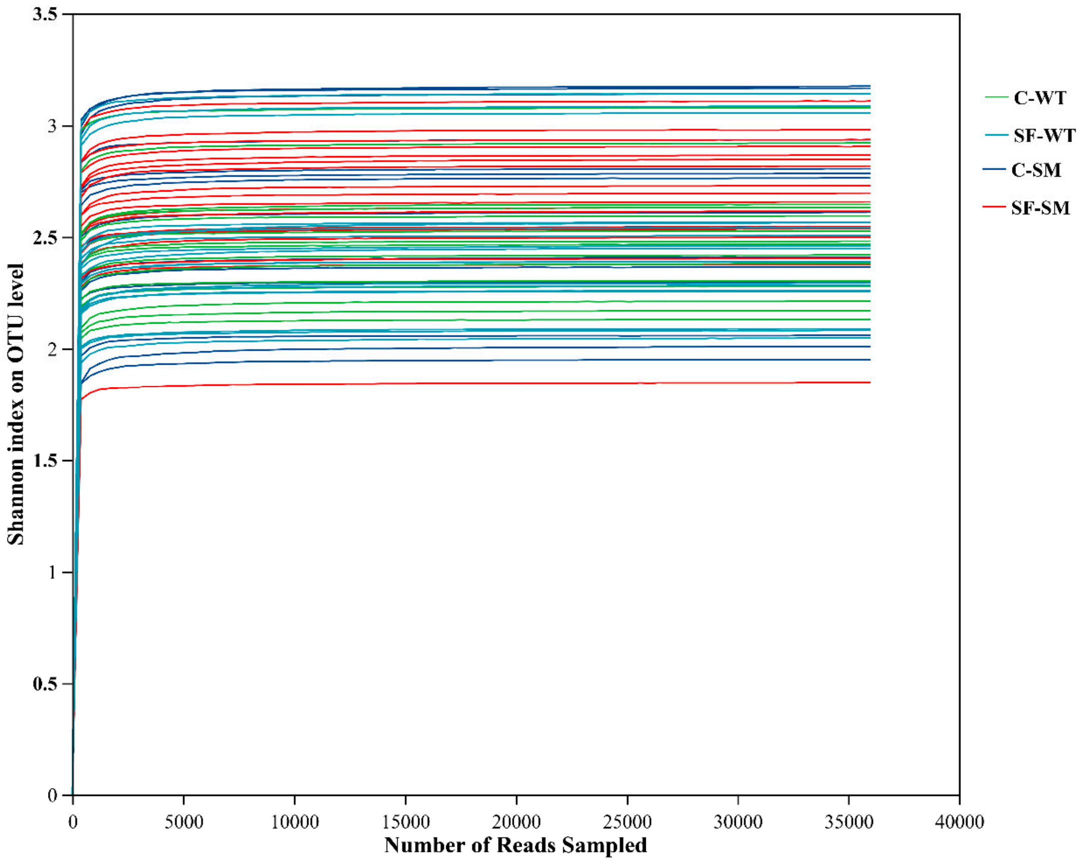

Figure S1. The bacterial rarefaction curves.

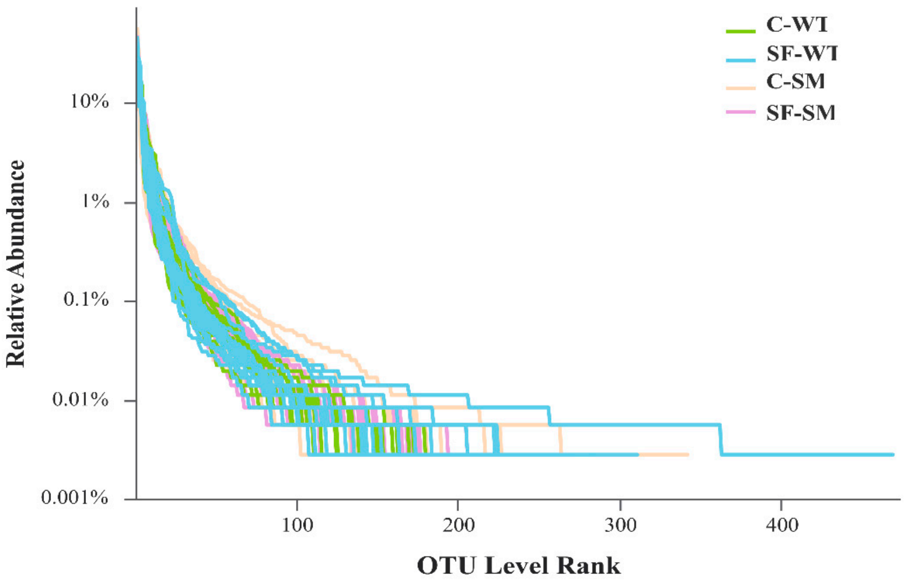

Figure S2. Bacterial rank abundance curves.

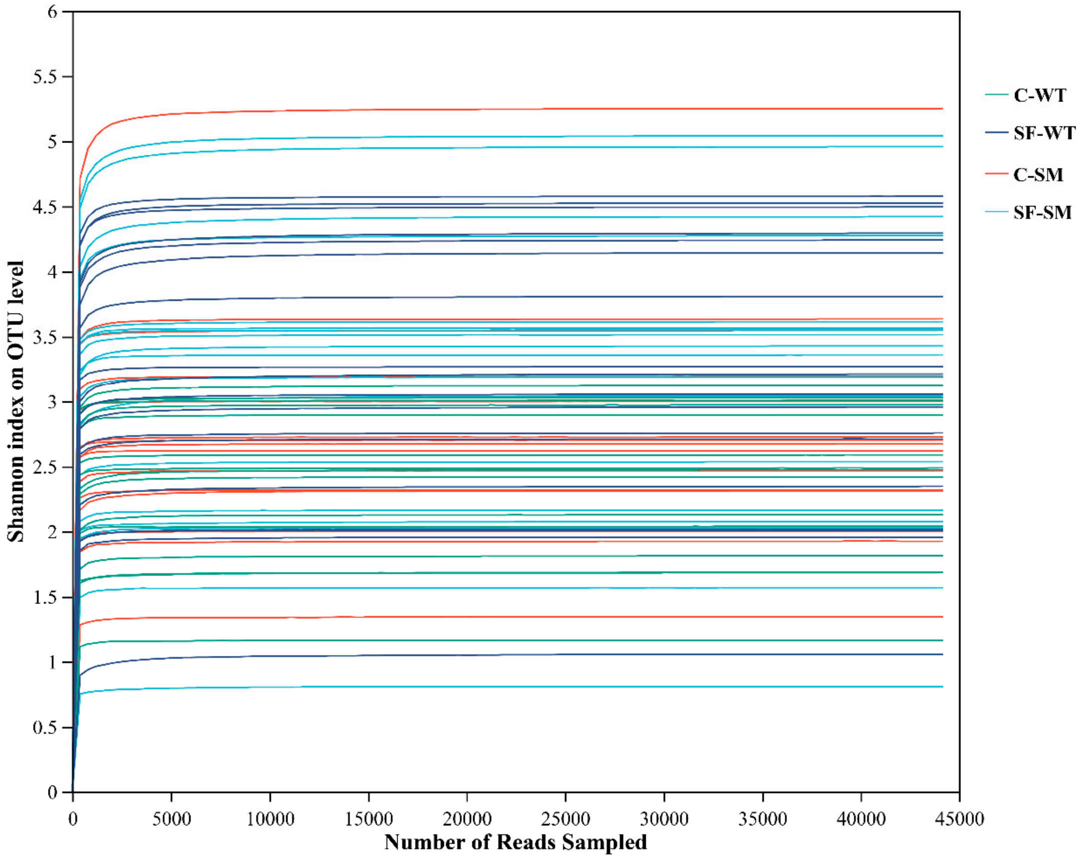

Figure S3. The Fungal rarefaction curves.

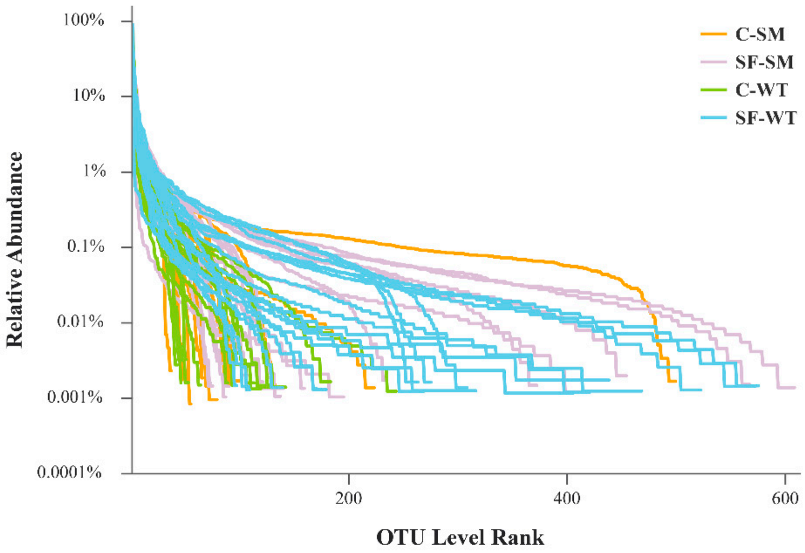

Figure S4. Fungal rank abundance curves.

Table S1. Table X Shapiro－Wilk Test for Normality of α- Diversity Indices (Bacteria and Fungi)

	Index	W-value	*P*-value
Bacteria	ACE	0.93607	0.00208
	Chao1	0.93114	0.001235
	Shannon	0.97783	0.2841
	Simpson	0.91263	0.0001979
Fungus	ACE	0.81610	＜0.0001
	Chao1	0.81539	＜0.0001
	Shannon	0.98194	0.4497
	Simpson	0.83720	＜0.0001

The Shapiro–Wilk test was used to assess the normality of the data. A *P*-value greater than 0.05 suggests that the data follow a normal distribution, whereas a *P*-value less than 0.05 suggests non-normality.

Table S2. Top ten enterobacterial phyla in terms of abundance in different combinations of seasons and environments

Bacteria/Phylum	C-WT	SF-WT	C-SM	SF-SM
Bacillota	82.43%	93.99%	63.31%	73.29%
Actinomycetota	8.89%	1.65%	15.52%	10.00%
Fusobacteriota	1.50%	1.43%	11.13%	8.11%
Pseudomonadota	5.60%	2.09%	6.32%	6.64%
Bacteroidota	1.41%	0.67%	3.49%	1.86%
Patescibacteria	0.02%	0.02%	0.09%	0.02%
Chloroflexi	0.03%	0.03%	0.06%	0.02%
Campylobacterota	0.10%	0.00%	0.02%	0.00%
unclassified k norank d Bacteria	0.03%	0.02%	0.05%	0.02%
Cyanobacteriota	＜0.01%	0.03%	0.01%	0.03%
others	＜0.01%	0.05%	0.01%	＜0.01%

Table S3. Top 20 Enterobacteriaceae genera in abundance in different seasonal and environmental combinations

Bacteria/Genus	C-WT	SF-WT	C-SM	SF-SM
Clostridium_sensu_stricto_1	27.46%	36.95%	7.84%	16.31%
Peptoclostridium	19.42%	5.31%	25.60%	15.48%
Paeniclostridium	11.26%	12.43%	3.51%	5.05%
Collinsella	8.07%	1.35%	12.64%	8.82%
Fusobacterium	1.50%	1.42%	11.11%	8.10%
Blautia	4.82%	3.95%	4.85%	8.26%
Peptoniphilus	1.10%	7.30%	1.81%	7.08%
Peptostreptococcus	1.91%	4.45%	4.40%	3.46%

Escherichia-Shigella	1.65%	0.68%	4.50%	4.48%
Ruminococcus_gnavus_group	3.16%	1.26%	2.38%	3.06%
Catenisphaera	2.13%	0.96%	3.49%	2.13%
Clostridium_sensu_stricto_7	1.34%	5.07%	0.38%	0.50%
Bacteroides	1.21%	0.51%	3.22%	1.60%
Lachnoclostridium	0.93%	1.11%	1.57%	2.69%
Clostridium_sensu_stricto_13	0.82%	2.20%	0.61%	0.97%
Clostridium_sensu_stricto_4	1.28%	1.90%	0.64%	0.69%
Romboutsia	0.15%	3.14%	0.11%	0.22%
Ruminococcus_torques_group	0.97%	0.74%	1.01%	0.86%
Streptococcus	0.58%	0.79%	0.83%	0.98%
Edwardsiella	2.77%	0.14%	0.15%	0.01%
others	7.48%	8.32%	9.35%	9.22%

Table S4. Top ten intestinal fungal phyla in terms of abundance in different combinations of seasons and environments

Fungus/Phylum	C-WT	SF-WT	C-SM	SF-SM
Ascomycota	56.93%	72.32%	48.07%	62.38%
Basidiomycota	39.69%	23.86%	42.13%	32.62%
unclassified k Fungi	1.55%	2.81%	5.58%	3.45%
Mortierellomycota	1.69%	0.43%	3.40%	0.35%
Chytridiomycota	0.08%	0.34%	0.19%	0.71%
Neocallimastigomycota	_	_	0.43%	_
Fungi_phy_Incertae sedis	0.00%	0.06%	0.01%	0.23%
Rozellomycota	0.06%	0.02%	0.03%	0.11%
Blastocladiomycota	＜0.01%	0.13%	0.01%	0.03%
Mucoromycota	＜0.01%	＜0.01%	0.06%	0.03%
others	＜0.01%	＜0.01%	0.10%	0.08%

Table S5. Top 20 genera of enteric fungi in terms of abundance in different seasons and environmental combinations

Fungus/Genus	C-WT	SF-WT	C-SM	SF-SM
Candida	22.28%	2.65%	6.99%	2.63%
Cutaneotrichosporon	8.19%	0.84%	12.43%	12.35%
Apiotrichum	10.89%	2.53%	15.22%	3.03%
Ascodesmis	0.01%	7.92%	11.14%	4.14%
Cystobasidium	6.95%	2.87%	4.22%	7.20%
Debaryomyces	8.30%	4.36%	5.53%	1.91%
unclassified _k__Fungi	1.55%	2.81%	5.58%	3.45%
Trichosporon	0.91%	8.15%	0.33%	2.44%
Fusarium	0.03%	5.42%	0.52%	5.23%
unclassified _o__Pleosporales	0.21%	6.37%	2.43%	1.10%
Periconia	0.78%	2.68%	1.54%	4.35%

Cladosporium	3.52%	1.88%	1.95%	1.72%
unclassified _f__Didymellaceae	3.09%	2.74%	0.68%	1.74%
Aspergillus	5.68%	0.28%	1.59%	0.49%
Rhodotorula	5.98%	0.74%	0.24%	0.81%
Diutina	_	0.03%	0.03%	5.58%
Epicoccum	＜0.01%	3.84%	0.08%	1.16%
Pseudopithomyces	0.02%	4.31%	_	0.38%
unclassified _o__Hypocreales	4.03%	0.40%	0.06%	0.07%
Bipolaris	0.06%	3.58%	0.03%	0.18%
others	17.53%	35.61%	29.41%	40.03%

Table S6. Intergroup comparison results for bacteria based on Adonis (with FDR correction)

Group	R^2	*P* value	adj *P* value
C-WT×C-SM	0.15576693	0.001	0.0020
C-WT×SF-WT	0.12851354	0.004	0.0060
C-WT×SF-SM	0.08695991	0.006	0.0072
C-SM×SF-WT	0.30682964	0.001	0.0020
C-SM×SF-SM	0.06766518	0.044	0.0440
SF-WT×SF-SM	0.20382667	0.001	0.0020

Table S7. Intergroup comparison results for fungi based on Adonis (with FDR correction)

Group	R^2	*P* value	adj *P* value
C-WT×C-SM	0.08798350	0.003	0.0036
C-WT×SF-WT	0.15073752	0.001	0.0015
C-WT×SF-SM	0.13296522	0.001	0.0015
C-SM×SF-WT	0.12357800	0.001	0.0015
C-SM×SF-SM	0.07254577	0.004	0.0040
SF-WT×SF-SM	0.07941876	0.001	0.0015
